# A New Surgical Technique for Internal Shoulder Contractures Secondary to Obstetric Brachial Plexus Injury: An Anterior Coracohumeral Ligament Release

**DOI:** 10.1055/s-0039-1693746

**Published:** 2019-08-13

**Authors:** C. Sarac, S. Hogendoorn, R.G.H.H. Nelissen

**Affiliations:** 1 Department of Orthopaedic Surgery, Leiden University Medical Center, Leiden, The Netherlands

**Keywords:** birth injuries, muscle skeletal surgery, brachial plexus injuried child, brachial plexus surgery

## Abstract

**Background** Obstetric brachial plexus injuries result from traction injury during delivery; 30% of these children sustain persisting functional limitations related to an external rotation deficit of the shoulder. The aim of this study was to compare the intraoperative gain in external rotation after a posterior subscapular release and an anterior coracohumeral ligament release.

**Methods** This is a prospective study on 102 children with an internal rotation contracture of the shoulder who received either a posterior subscapular release (posterior skin incision along the medial border of the scapula of 3–5 cm) or an anterior (5-mm skin incision) coracohumeral ligament release between 1996 and 2010. After general anesthesia, internal and external rotations in both adduction and abduction were measured before and after the surgical release.

**Results** After a posterior subscapular release, the intraoperative external rotation improved with a mean of 64 degrees (95% confidence interval [CI]: 54–74; *p* < 0.001) in adduction and with a mean of 41 degrees (95% CI: 32–49; *p* < 0.001) in abduction. After an anterior coracohumeral ligament release, external rotation increased with a mean of 61 degrees (95% CI: 56–66; *p* < 0.001) in adduction and a mean of 42 degrees in abduction (95%CI: 39–45, *p* < 0.001). Differences between these two groups were not statistically different.

**Conclusion** The anterior release technique shows comparable results with the posterior subscapular release. And since it is performed through a smaller incision of 5 mm, this is our preferred method to increase passive external rotation.

**Level of evidence** II.

## Introduction

Obstetric brachial plexus injuries (OBPIs) are a result of a traction injury during delivery. The incidence of OBPI varies from 0.38 to 5.1 per 1,000 live births in various countries. 1 2 The majority of OBPIs are mild, and spontaneous functional recovery will be present in approximately 70% of children between 4 and 6 months of age. The remaining 30% are left with functional deficits. 3

The ultimate purpose of treatment in OBPIs is optimal restoration of arm and hand function. Depending on the extent of the brachial plexus injury, either conservative therapy (i.e., contracture preventive therapy) or nerve surgery in more severe lesions may improve this upper extremity function. But after this initial treatment, a growing child might again have recurring functional deficits, and anatomical changes may occur, limiting functionality. 4 5 6 7 8 9 10 One of these sequelae is limited external rotation of the shoulder, limiting hand positioning to the mouth and head. This internal rotation contracture of the shoulder, when limiting arm functionality, is an indication for secondary surgery (internal contracture releases in combination with tendon transfers to promote active external rotation). 11 12 13 Until now, most studies describe extensive surgical procedures or muscle release procedures such as the posterior subscapular slide or an anterior subscapularis tendon lengthening. However, shoulder balance between external and internal rotations can be restored by only a subtle anterior coracohumeral release, thus leaving the subscapular muscle intact. Since outcome in these heterogeneous brachial plexus injury patients shows great variability on active range of motion outcome, we aimed to compare only the passive gain in external rotation in two planes between an anterior coracohumeral release and the classic posterior subscapular release.

## Methods

All OBPI children at our institution who had an orthopaedic surgical intervention for an internal rotation contracture of the shoulder were prospectively entered in a database as of 1995.

This study involves all children who had an internal rotation contracture of the shoulder between 1996 and 2010, for which only an internal rotation contracture release without tendon transfers around the shoulder was performed. Two types of internal contracture releases were performed in two successive time periods. The posterior release (subscapular slide), as described by Carlioz and Brahimi, 14 was performed between 1996 and 2001. Due to recurrence of contractures, between 2001 and 2002, an extensive anterior release with subscapular tendon lengthening was performed, and since the latter caused an external rotation contracture, the technique was adopted. As of 2002, an anterior shoulder technique was performed using a 5-mm-long skin incision, releasing the coracohumeral ligament. All surgeries were performed by the senior author.

A total of 128 children underwent an internal contracture release during this period, of which 26 had a posterior subscapular slide and 76 only an anterior coracohumeral ligament release ( Table 1 ). The remaining 26 patients were operated in the transition from the posterior to anterior release and received a combination of a posterior and anterior release and were therefore not analyzed in detail.

**Table 1:** Patient characteristics

Patient's characteristics	Anterior release, *N* = 76	Posterior release, *N* = 26	Combined posterior and anterior release, *N* = 26
Gender, male:female	36:40	17:9	12:14
Affected side, left:right	30:46	14:12	11:15
Median age (lower quartile; upper quartile)	4(3; 6)	3 (SD: 2; 4)	3 (2; 5)
Type of lesion			
C5-C6	51	18	18
C5-C7	22	5	5
C5-C8	1	1	1
C5-T1	1	2	2
Unknown	1	–	–

Abbreviation: SD, standard deviation.

### Surgical Techniques

All operations were performed under general anesthesia. After general anesthesia, all shoulder range of motion values—internal and external rotation in both adduction and abduction—were measured before and after the internal contracture release procedure by the surgeon.

#### Posterior Subscapular Slide

In 26 children, a 3- to 4-cm-long incision along the inferior angle and the lateral border of the scapula was used to expose the latissimus dorsi teres major interval, in this interval, the inferior one-third of the medial scapular border was identified and incised with diathermiccautery. The subscapularis was then released posteriorly from the scapula with a raspatorium.

#### Anterior Coracohumeral Ligament Release

**Fig. 1:**
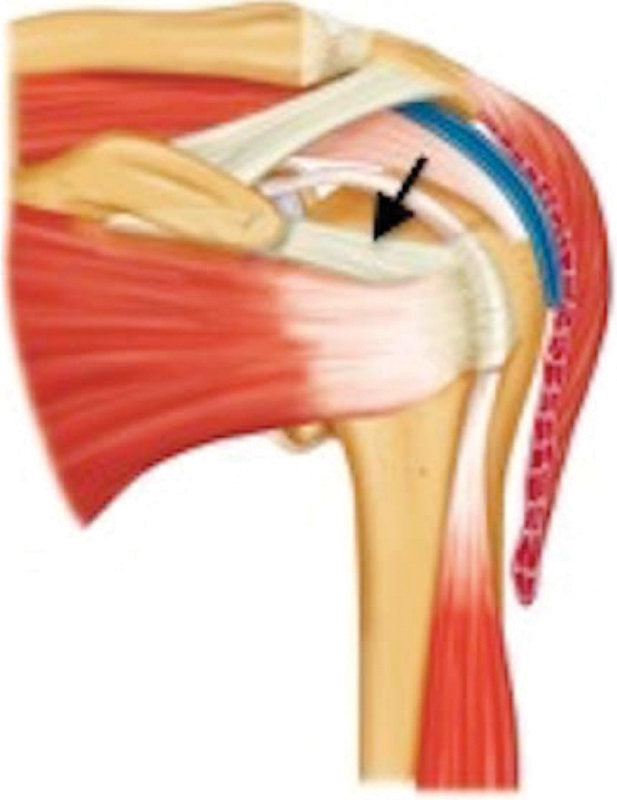
Anatomy of the shoulder ( *arrow* indicates the coracohumeral ligament).

A deltopectoral incision of 5 to 7 mm was performed to expose the coracoid in 76 children. After identification the coracoid, the conjoined tendon was moved laterally, a small 3-mm-wide hook was placed underneath the coracoacromial ligament, and the base of the coracoid was visualized. Then an incision was made at the base of the coracoid, with a no. 15 blade, releasing the coracohumeral ligament ( Fig. 1 ) at the anterior capsule of the shoulder at a length of ∼3 mm.

### Statistical Analysis

A paired *t* -test was used to analyze the intraoperative gain in external rotation for both groups. An unpaired *t* -test was used to compare the intraoperative gain in external rotation for both groups.

## Results

### Posterior Subscapular Release

Intraoperative passive external rotation increased with 64 degrees (95% confidence interval [CI]: 54–74; *p* < 0.001) ( Table 2 ) in adduction and 41 degrees (95% CI: 32–49; *p* < 0.001) in abduction ( Table 3 ).

**Table 2:** Pre- and postincisional ER in adduction in both groups

Surgical technique	Preincisional ER in adduction, median (lower quartile; upper quartile)	Postincisional ER in adduction, median (lower quartile; upper quartile)
Posterior subscapular release	–5 (–28; 14)	60 (50; 70)
Anterior coracohumeral ligament release	–10 (–30; 0)	50 (40; 60)

Abbreviation: ER, external rotation.

**Table 3:** Pre- and postincisional ER in abduction in both groups

Surgical technique	Preincisional ER in abduction (median, lower quartile; upper quartile)	Postincisional ER in abduction (median, lower quartile; upper quartile)
Posterior subscapular release	40 (30;60)	88 (80;90)
Anterior coracohumeral ligament release	40 (30;45)	80 (75;80)

Abbreviation: ER, external rotation.

#### Anterior Coracohumeral Release

Intraoperative passive external rotation increased with 61 degrees (95% CI: 56–66; *p* < 0.001) ( Table 2 ) in adduction and with 42 degrees (95% CI: 39–45; *p* < 0.001) in abduction ( Table 3 ).

#### Comparison between Both Groups

Comparison between the anterior coracohumeral release and the posterior subscapular release showed that improvement in external rotation in both adduction ( *p* = 0.08) and abduction ( *p* = 0.17) were comparable in both groups.

## Discussion

All patients gained sufficient external rotation after both the posterior subscapular glide technique and the limited anterior coracohumeral ligament surgical internal rotation contracture release procedures. The goal of both techniques is to enable active muscle action around the shoulder joint to enable active external rotation, thus allowing hand-to-mouth and hand-to-head movements. Soft tissue release techniques can have side effects due to overbalancing the released movement of action, thus creating an external rotation contracture, which also limits functionality due to obstructing hand-to-belly movements. A release procedure is a delicate balancing procedure, allowing functionality of the arm if performed correctly. Several methods of tendon lengthening or treating a contracture exist. Most studies describe procedures including a posterior subscapular sliding technique releasing the internal rotation contracture, which also results in an increase in global function. 15 However, releasing the subscapular muscle from the ventral scapular blade can result in early recurrence of the internal rotation contracture, as found by us and others. 16 Even more, this muscle sliding technique of the subscapular muscle from the scapula might damage the subscapular muscle. Since the subscapular muscle is one of the prime movers of the shoulder, this might have an effect on overall shoulder function. The presence of recurrences and potential muscle damage urged us to start a different contracture release technique. The technique originated from an open anterior subscapular lengthening technique with resection of the coracoid process as described by Birch. 17 During these procedures, it was observed that during a stepwise release of the anterior tissue with control of the range of motion, external rotation during each step of this release showed an increase in the range of motion. The major influence on external rotation was the release of the coracohumeral ligament while leaving the coracoid process and the subscapular muscle intact. The coracohumeral ligament has been described as a constraint in frozen shoulders, limiting external rotation. Another study described an anterior capsular release while sparing the subscapular muscle. However, this study included only a small group of patients ( *n* = 14), and the release was performed in combination with a latissmus dorsi transfer, 18 which also has an effect on an internal rotation contracture. Thus results may have been influenced by the detachment and subsequent transfer of the tendon. Our study is in a larger group, and only the intraoperative effect of the release procedure is described, which could not be influenced by the combined release and detachment of other shoulder internal rotators such as the latissimus dorsi or teres major. Other studies describing an arthroscopic anterior release combine this procedure with a subscapular tenotomy. We believe that damage to the subscapular muscle interferes with functionality of the shoulder since the subscapular muscle is one of the prime movers of the shoulder, thus comprising not only internal rotation movement but also overall shoulder function (i.e., abduction/elevation). 19 20

The less invasive anterior release technique of the coracohumeral ligament is currently still the preferred soft tissue release at our center, and we have not performed a subscapular lengthening procedure since 2001. As mentioned in the Introduction section, we did not describe postoperative follow-up since the aim of the study was to describe a surgical technique.
